# Supplementary material for: Molecular Characterization of Small Ruminant Lentiviruses in Polish Mixed Flocks Supports Evidence of Cross Species Transmission, Dual Infection, a Recombination Event, and Reveals the Existence of New Subtypes within Group A
Source: Viruses. 2021 Dec 16;13(12):2529. doi: 10.3390/v13122529 (PMC8708130; doi:10.3390/v13122529)
Supplement: Supplementary file 1 [file viruses-13-02529-s001.zip › viruses-1484523-supplementary.pdf]

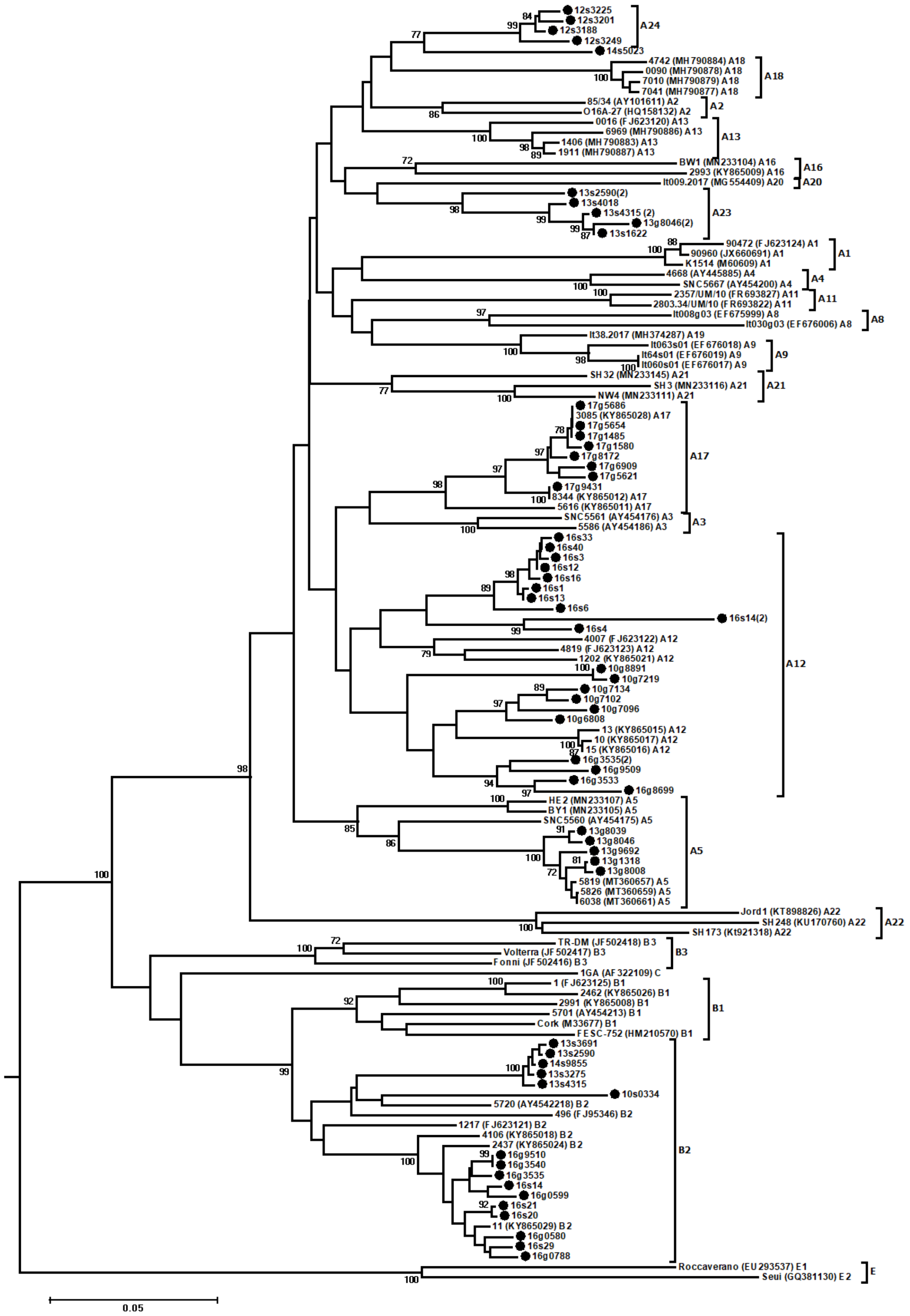

Figure S1. Neighbor-joining phylogenetic tree based on alignment of CA fragment of *gag* gene. Sequences from this study are labeled by black circles and their names are proceeded by the flock origin and the animal species (s-sheep; g-goat). Numbers at the branches indicate the percentage of bootstrap values obtained from 1000 replicates. Bootstrap values >70% are shown.

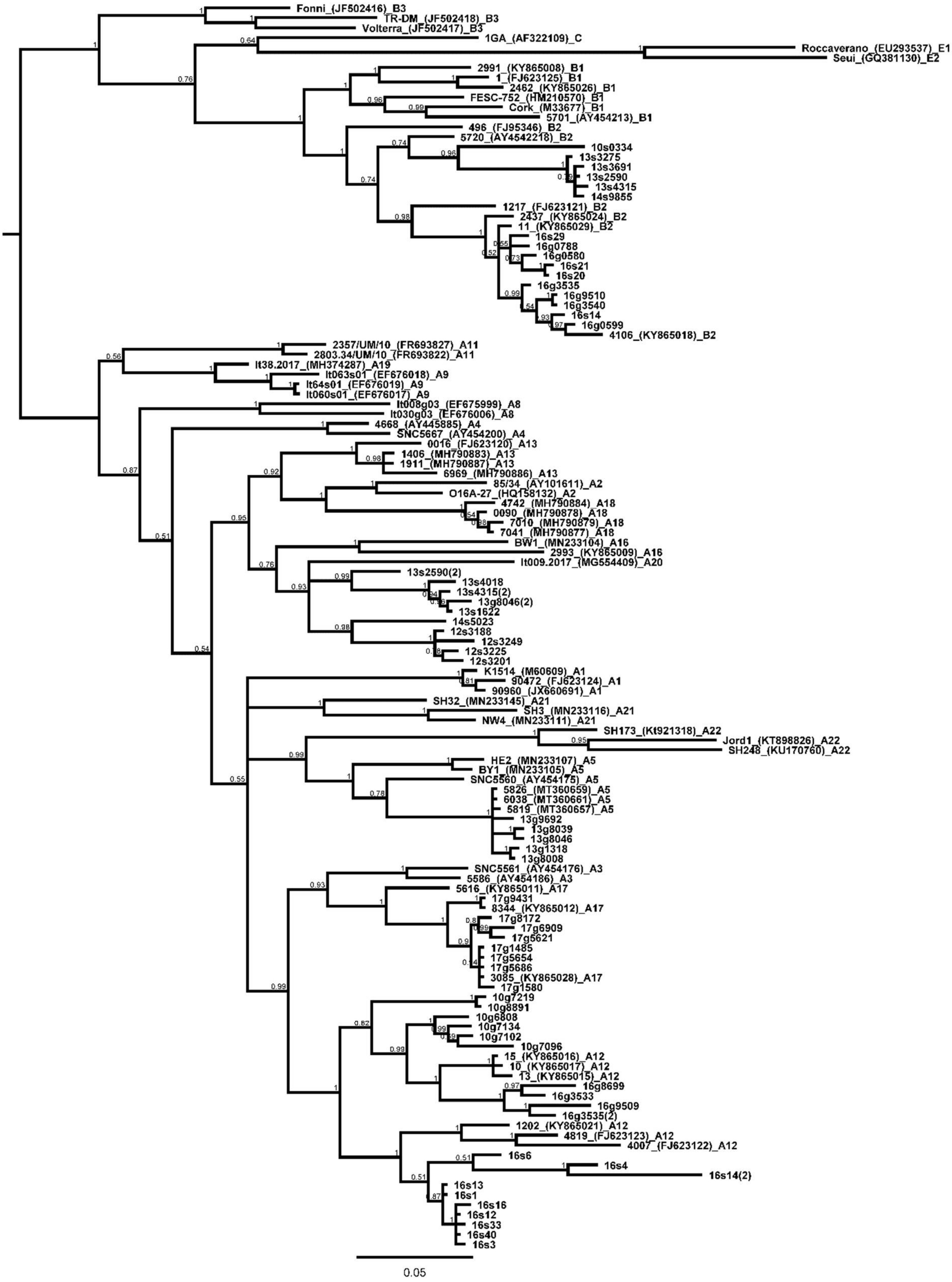

Figure S2. Bayesian phylogenetic tree based on alignment of CA fragment of *gag* gene. The name of sequences from this study are proceeded by the flock origin and the animal species (s-sheep; g-goat). Posterior probability of each node is showed above branches.

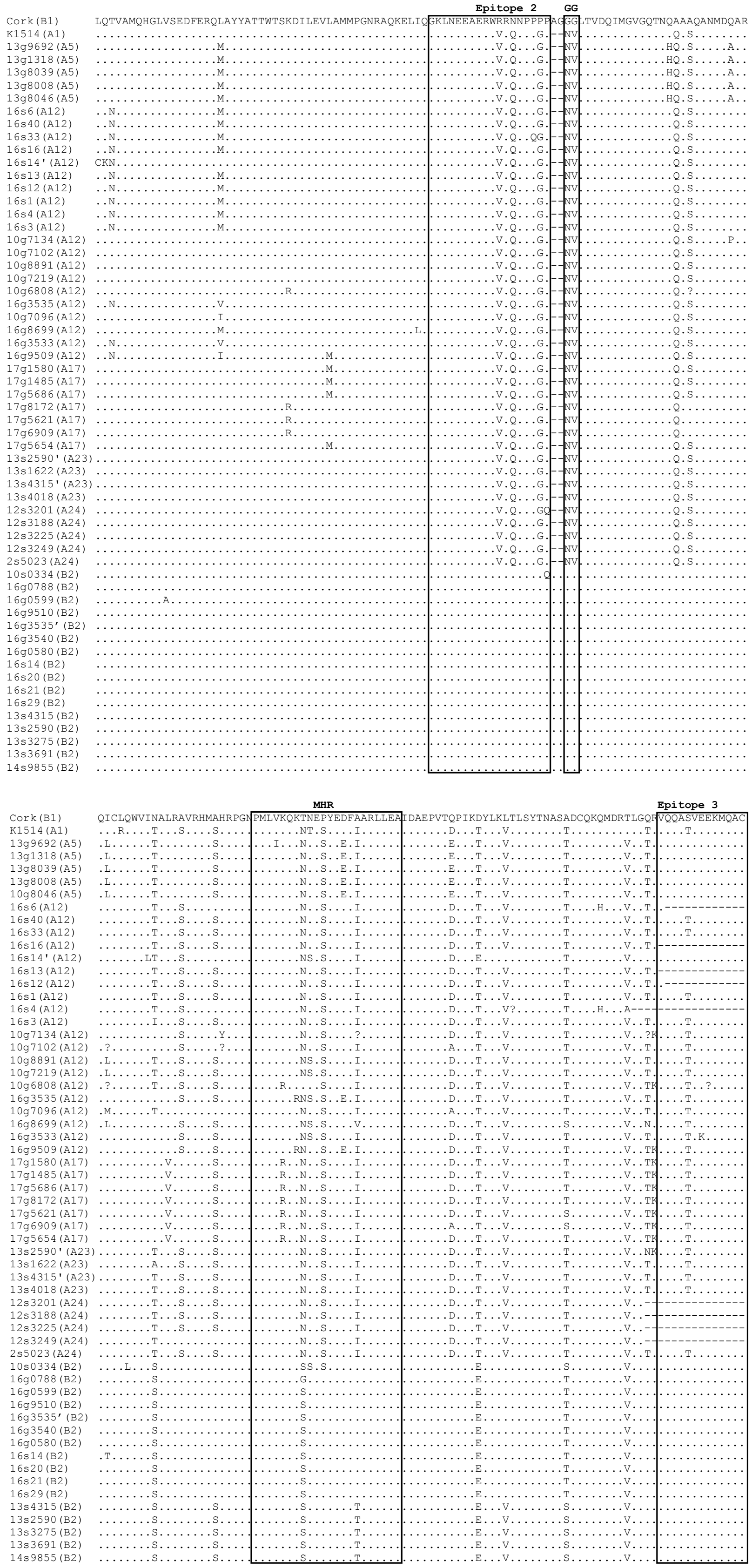

Figure S3. Alignment of deduced amino acid sequences of immunodominant epitopes of capsid protein and major homology region (MHR) of the SRLVs obtained in this study and reference strains. Identical residues are indicated by dots (.).
